# Sleeplessness

**Published:** 1885-04

**Authors:** 


### Sleeplessness.

A foreign exchange very truly says that sleeplessness is really wakefulness, and that before we resort to stupefying drugs to remedy this condition, we should inquire into the cause and remove it if possible. The cases of Napoleon and other busy men, who develop the power of self-induced sleep to such an extent as to be able to rest whenever aud wherever they please, for longer or shorter intervals, as the conditions admitted, are quoted. We know that excessive mental work, worry, the abuse of alcoholic beverages or anything that will tend to keep too much blood in the brain, will ultimately produce a chronic engorgement of that organ that will render physiological sleep almost an impossibility. We have known insomnia, due to excessive alcoholic indulgence, to be very effectually relieved (and permanently so) by the application of dry cups to the nape of the neck. To discover the cause, will, of course, in many cases, take time, and involve a scientific testing of the relative excitabilities of the sense-organs, central or radial and peripheral. The discovery of the cause, however, affords ample recompense for the trouble of searching for it. With the sphygmograph and a few test appliances, such as Galton's whistle, an optometer, and other instruments, the recognition of the form and cause of sleeplessness can be made in a brief space, and then, and then *only* we protest, it can be scientifically—i. e., physiologically—treated.

